# Buffalo Medical Association

**Published:** 1881-05

**Authors:** 


### Society Reports.

### BUFFALO MEDICAL ASSOCIATION.

*Stated Meeting, April 5, 1881.*

DR. HOWE, IN THE CHAIR, AND DR. MACNIEL, SECRETARY.

Present, Drs. Hauenstein, White, Samo, Rochester, Trowbridge, Nichell, Davidson, Cronyn, Johnson, Barker, Moody, O'Brien, Brecht, Green, Hartwing, Briggs, Bartow and Keene.

The first paper presented was on the treatment of spinal curvature by Dr. Bartow. The writer reviewed briefly those causes of the disease which might be removed by mechanical appliances, and then showed how the various indications could be fully carried out by the use of a properly adjusted leather brace. In the discussion which followed, the method proposed received very favorable criticism, especially from those who had witnessed its working. The paper will be given entire, or in part in a future number of this Journal.

This communication was followed by one from Dr. S. S. Green, on the treatment of membranous croup by the local use of perchloride of iron. The histories of seven cases were given, in which a concentrated solution of this salt had been thrown, in an atomized form, into the larynx, and, of the number, there were two deaths with five recoveries.

The importance of the subject and the variety of opinion concerning different modes of treatment, rendered it advisable to postpone extended discussion till another meeting, and this was accordingly done.

The following resolutions, presented by Dr. James P. White, were adopted by unanimous vote. They have already been published elsewhere, but it seems well to place them on record here.

*Whereas*, It is the duty of the members of this Society to indicate any existing sources of danger to the public health; and whereas, in the opinion of this Society the streets of this city are in a very filthy and unhealthy condition, thereby greatly endangering the health of the inhabitants; therefore

*Resolved*, That, as guardians in sanitary matters, we feel it incumbent on us to call the attention of the city authorities to the filthy state of the streets, and urge their atttention to this most important subject as soon as practicable.

*Resolved*, That we have been pleased to notice the suggestions of the Health Physician recently made to the Common Council, and earnestly commend them to their careful consideration.

*Resolved*, That a committee of thirteen be appointed from the members of this Society to act in concert with the Health Physician in urging some definite action in these important sanitary measures.

*Resolved*, That a copy of these proceedings be sent to the Common Council and to the daily papers for publication.

Dr. Barker, offered a resolution endorsing "an act to regulate the practice of pharmacy and the licensing of persons to carry on such practice, and the sale of poisons," which act is now pending before the Legislature. The motion to adopt this resolution was also carried.

The Assocation then listened to the address of Dr. Howe, which was substantially as follows:

"Even if it were not customary for the retiring President of Scientific Associations like this, to offer, at least, a few suggestions, I should be partly compelled to do so, by that clause of the By-Laws which constitutes me *ex-officio* chairman of the Board of Trustees. For during the past year, as in the one preceeding, this Board has made special effort to have the increasing zeal of the members manifested by the satisfactory character of the work accomplished, and we can now look at the result, with a pardonable feeling of self-congratulation. Soon after the annual election in 1879, a move was made toward the adoption of a new constitution, which offered decided advantages over the regulations previously governing the Association. Among other changes thus introduced, was the selection, several months in advance, of the subjects of papers to be prepared, and as a consequence, some phase of a medical topic has been presented and intelligently discussed at each meeting. Indeed, an examination of the existing records of the Association, since its incorporation in 1856, shows that seldom, if ever, has there been either as large an average attendance, or such a succession of interesting papers as during the year past. Satisfactory as this may be, compared with a former state of affairs, it must however be acknowledged, with regret, that there are very many and very important improvements which might yet be made. It is unfortunate that the meetings are held at a place, rather difficult of access, and while the members feel under very great obligations to the Faculty of the College for the courtesies offered, most of them appreciate the advantages of a more centrally located and agreeable place of meeting. It should not be understood, that the utility of the morbid specimens here collected is overlooked, and next in importance to the procuring of suitable accommodations, the Board would recommend that some steps be taken toward the establishment of a museum, which, by gradual additions, would ultimately become highly instructive. Should a committee be appointed to report on this last recommendation, we would suggest that they also take into consideration the advisability of forming, with the museum, a collection of such standard works and periodicals, as shall constitute the nucleus of a future medical library. Some special effort should, furthermore, be made to concentrate into one energetic society all that zeal for professional advancement which is now too frequently expended in aimless directions. Indeed, when we consider how many practitioners there are in Buffalo who count themselves as real students, how many who imagine themselves working for the best good of their patients and, yet, how comparatively few important observations are recorded, or discoveries are made; when we remember this, we are forced to confess that the standard of professional excellence is not even yet as high as it might easily be made. It is a pleasure, therefore, to be able to chronicle any signs of progress, or any efforts which tend to produce a more healthy tone. Finally, I have to thank the Association for the honor of being chosen, for two consecutive years, as the one to preside over its deliberations. An examination of the records fails to show that any one of my predecessors has occupied the same position more than one term at a time. I appreciate, therefore, the kindly spirit and continued forbearance of the members, and congratulate myself that I have taken any part in an organization, which was deemed by its eminent founders, so conducive to professional harmony and so necessary for the advancement of medical science in this vicinity.

The proceedings closed with the election of officers for the ensuing year, which resulted in the choice of the following: For President, Dr. A. M. Barker; Vice-President, Dr. A. R. Davidson; Secretary, Dr. D. Macniel, and Treasurer, Dr. F. E. L. Brecht.

